# Supplementary material for: Bilophila wadsworthia aggravates high fat diet induced metabolic dysfunctions in mice
Source: Nat Commun. 2018 Jul 18;9:2802. doi: 10.1038/s41467-018-05249-7 (PMC6052103; doi:10.1038/s41467-018-05249-7)
Supplement: Supplementary file 1 — Supplementary Information [file 41467_2018_5249_MOESM1_ESM.pdf]

Supplementary Information

Supplementary Figures 1-9

Bilophila wadsworthia aggravates high fat diet induced metabolic dysfunctions in mice

Jane M. Natividad, Bruno Lamas, Hang Phuong Pham, Marie-Laure Michel, Dominique Rainteau, Chantal Bridonneau, Gregory da Costa, Johan van Hylckama Vlieg, Bruno Sovran, Celia Chamignon, Julien Planchais, Mathias L. Richard, Philippe Langella, Patrick Veiga, and Harry Sokol

Supplementary Figure 1

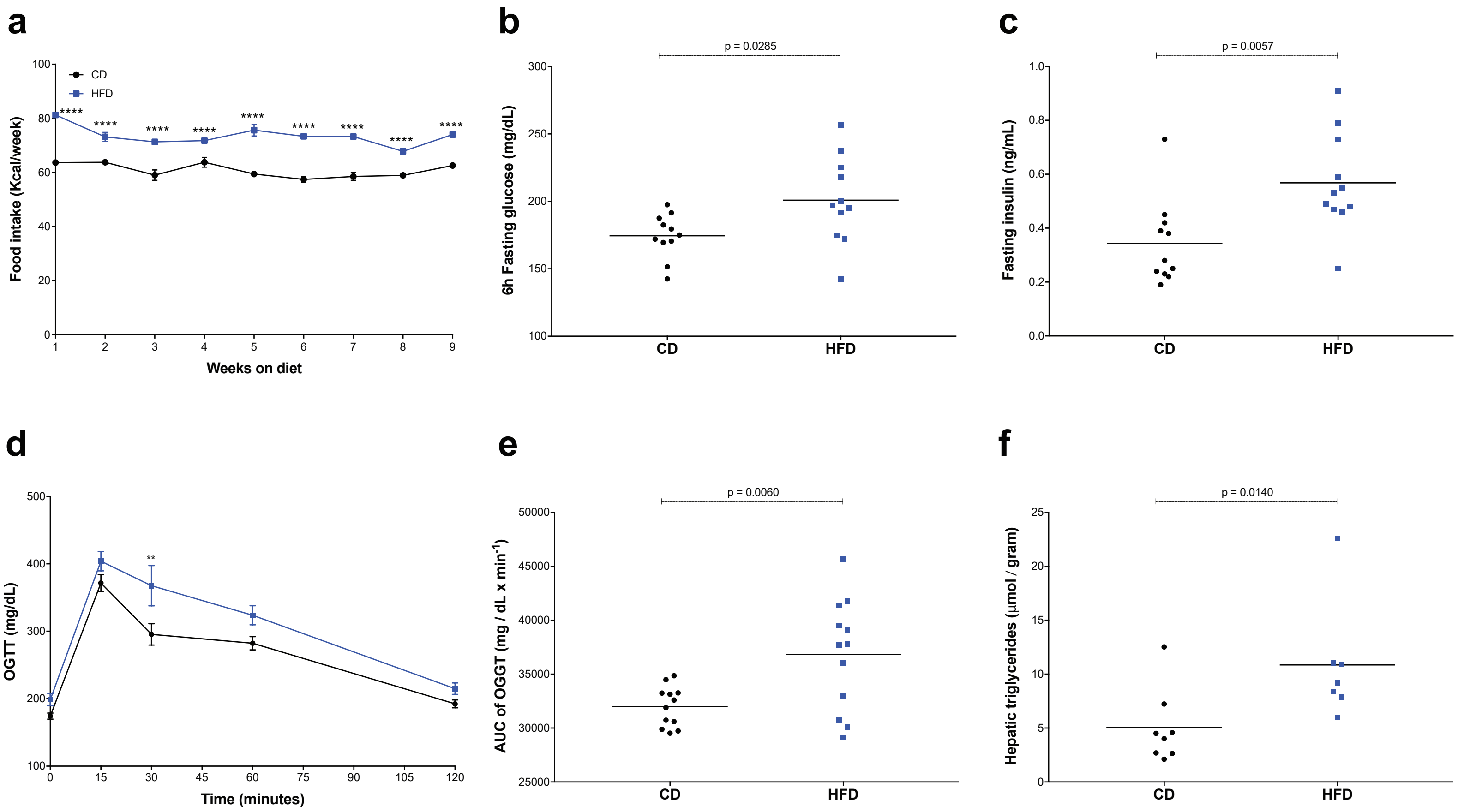

Supplementary Figure 1. Effect of HFD on *B. wadsworthia* intestinal load and metabolic host functions after 9 weeks. (**a**) weekly food intake of mice (n=10/group). (**b**) Blood glucose, (**c**) insulin, (**d**) Blood glucose level before and after oral glucose tolerance challenge (OGTT; 2g/kg mouse; n=10/group). (**e**) Area under the curve (AUC) of OGTT. (**f**) Liver triglycerides after 6h of food deprivation. Statistical comparison was performed by first testing normality using Kolmogorov-Smirnov test and then unpaired *t*-test or Mann-Whitney test. (*$p<0.05$, **$p<0.01$, ***$p<0.001$, ****$p<0.0001$). Error bars represents SEM.

Supplementary Figure 2

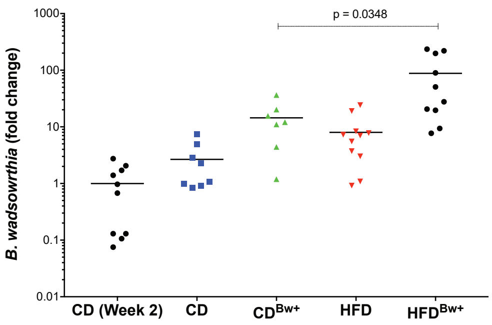

Supplementary Figure 2. *B. wadsworthia* intestinal load and host effects in mice fed with control diet and deliberately inoculated with *B. wadsworthia*.

Fold change of *B. wadsworthia* in mice fed with control diet (CD) or high-fat diet (HFD) relative to the load before *B. wadsworthia* inoculation (week 2). Statistical comparison was performed by first testing normality using Kolmogorov-Smirnov test and then unpaired *t*-test or Mann-Whitney test.

Supplementary Figure 3

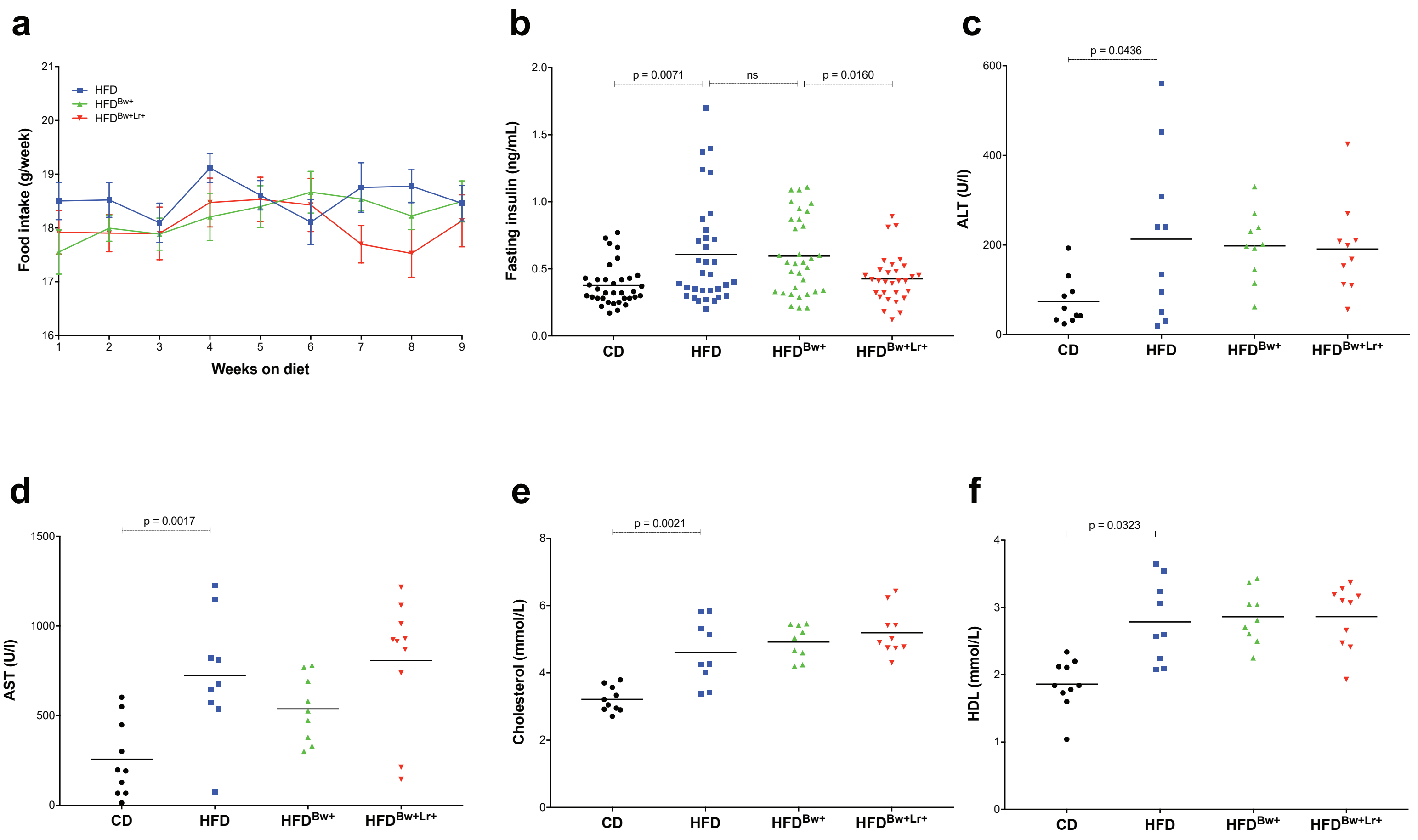

Supplementary Figure 3. Effect of *L. rhamnosus* CNCM I-3690 in mice fed with HFD and deliberately inoculated with *B. wadsworthia*. (**a**) Food intake (n=10/group). (**b**) Insulin, Concentration of (**c**) alanine transaminase (ALT), (**d**) aspartate transaminase (AST), (**e**) cholesterol, and (**f**) high-density lipoprotein after 6h of food deprivation. Statistical comparison was performed by first testing normality using Kolmogorov-Smirnov test and then unpaired *t*-test or Mann-Whitney test. Error bars represents SEM.

Supplementary Figure 4

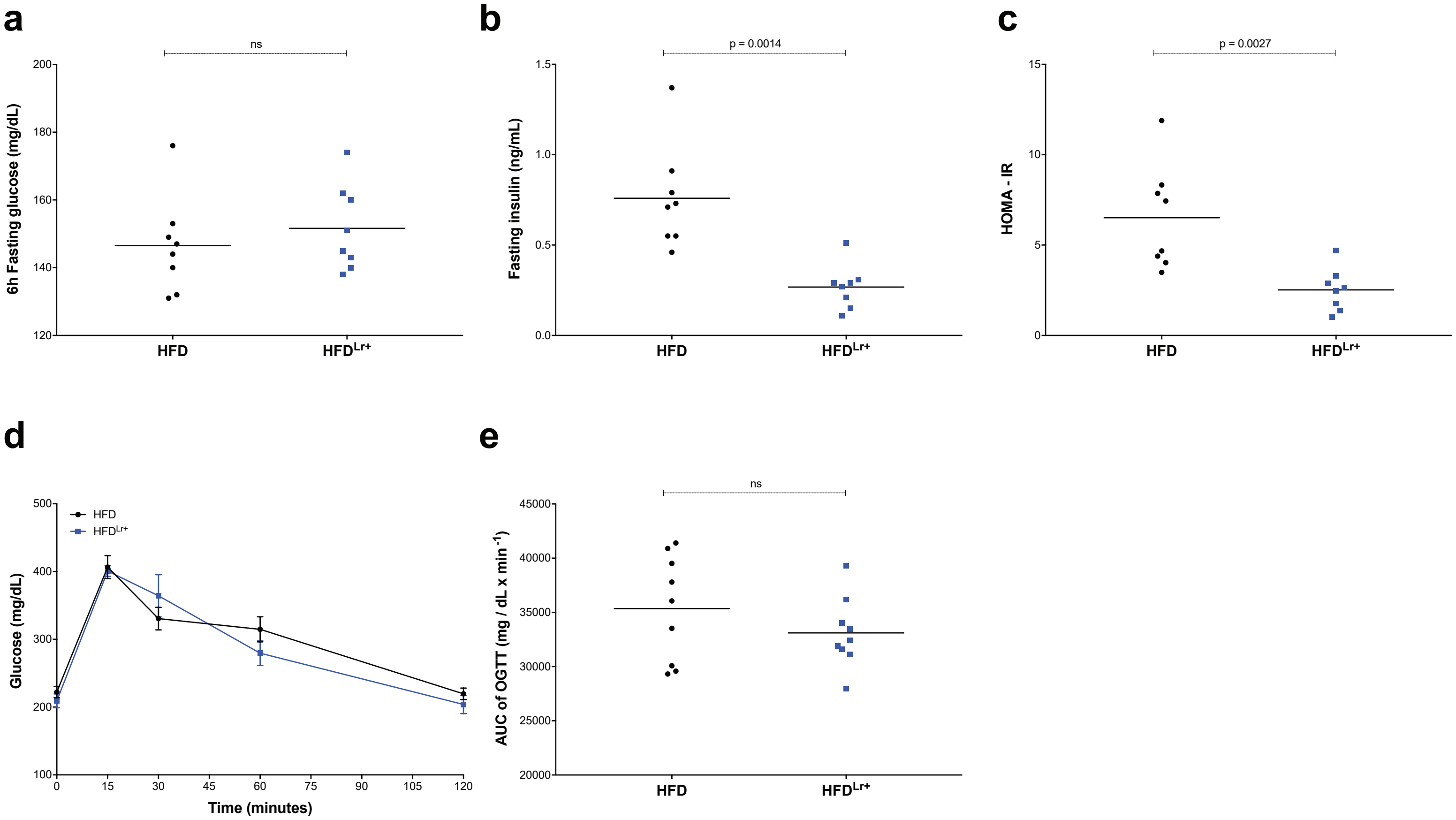

Supplementary Figure 4. Effect of *L. rhamnosus* CNCM I-3690 in HFD-fed mice. (**a**) Glucose, (**b**) insulin, (**c**) homeostatic model assessment-insulin resistance (HOMA-IR) after 6h of fasting. (**d**) Blood glucose level before and after oral glucose tolerance challenge (OGTT; 2g/kg mouse; n=9/group). (**e**) Area under the curve (AUC) of OGTT. Statistical comparison was performed by first testing normality using Kolmogorov-Smirnov test and then unpaired *t*-test or Mann-Whitney test. Error bars represents SEM.

Supplementary Figure 5

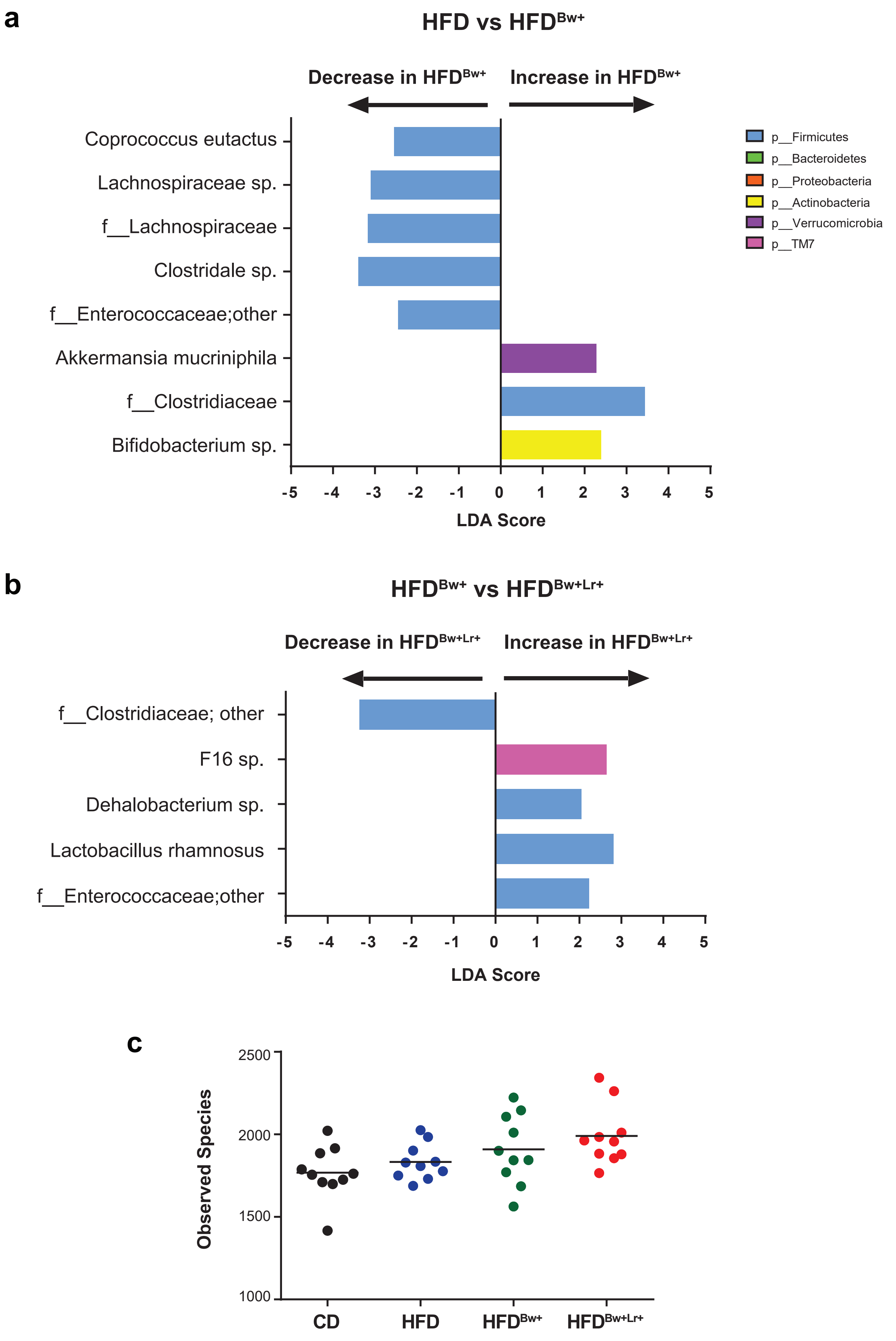

Supplementary Figure 5. Microbiota compositional profile of mice HFD-fed mice inoculated with or without *B. wadsworthia* and/or *L. rhamnosus* CNCM I-3690. (**a**) Discriminant analysis (LEFSE) of the fecal microbiota between HFD and HFD^{Bw+} group. (**b**) Discriminant analysis (LEFSE) of the fecal microbiota between HFD^{Bw+} and HFD^{Bw+Lr+} group. (**c**) Fecal microbiota alpha diversity, evaluated by Shannon index. Statistical comparison was performed by first testing normality using Kolmogorov-Smirnov test and then ANOVA or Kruskal-Wallis test with Bonferroni or Dunn's *post hoc* test. Error bars represents SEM.

Supplementary Figure 6

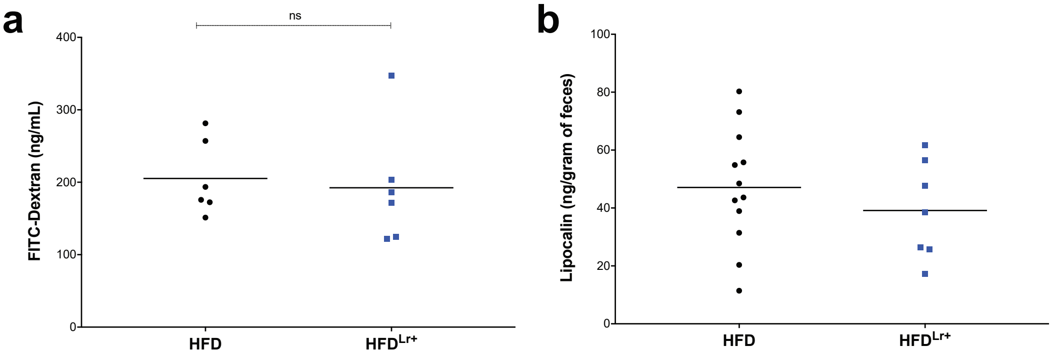

Supplementary Figure 6. Effect of *L. rhamnosus* CNCM I-3690 in HFD-fed mice. (**a**) Concentration of FITC-dextran in the serum 3h post-gavage. (**b**) Concentration of lipocalin-2 in the feces. Statistical comparison was performed by first testing normality using Kolmogorov-Smirnov test and then unpaired *t*-test or Mann-Whitney test. Error bars represents SEM.

Supplementary Figure 7

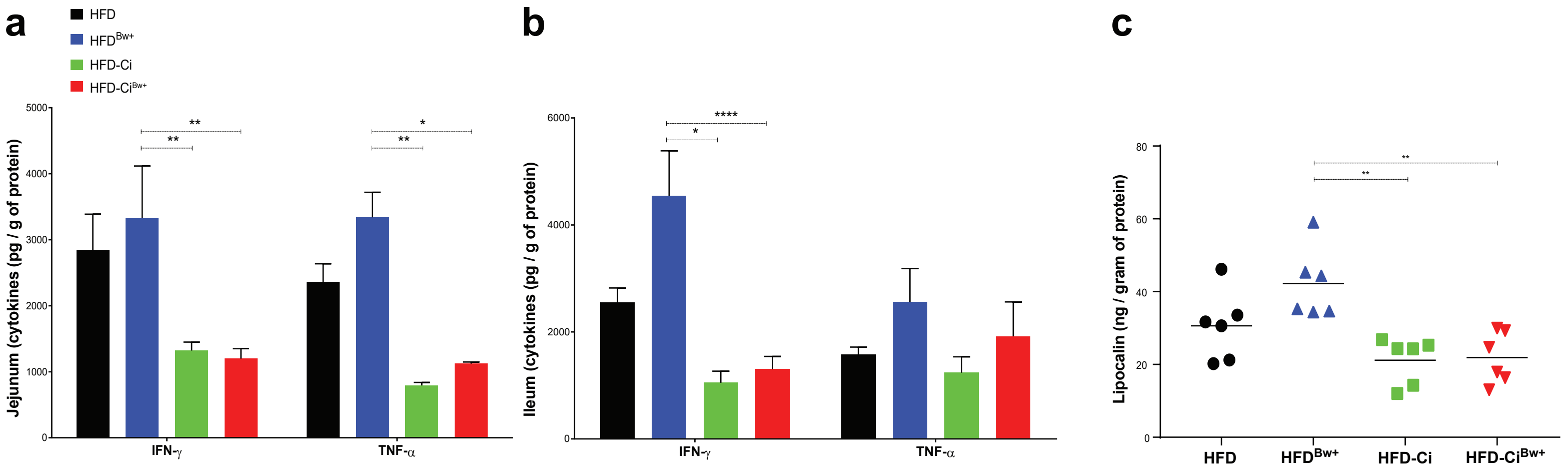

Supplementary Figure 7. Ciclosporine suppresses intestinal inflammation in HFD-fed mice. Cytokines level in (**a**) jejunal and (**b**) ileal homogenates. (**c**) Concentration of lipocalin-2 in the feces. Statistical comparison was performed by first testing normality using Kolmogorov-Smirnov test and then unpaired *t*-test or Mann-Whitney test. Error bars represents SEM.

Supplementary Figure 8

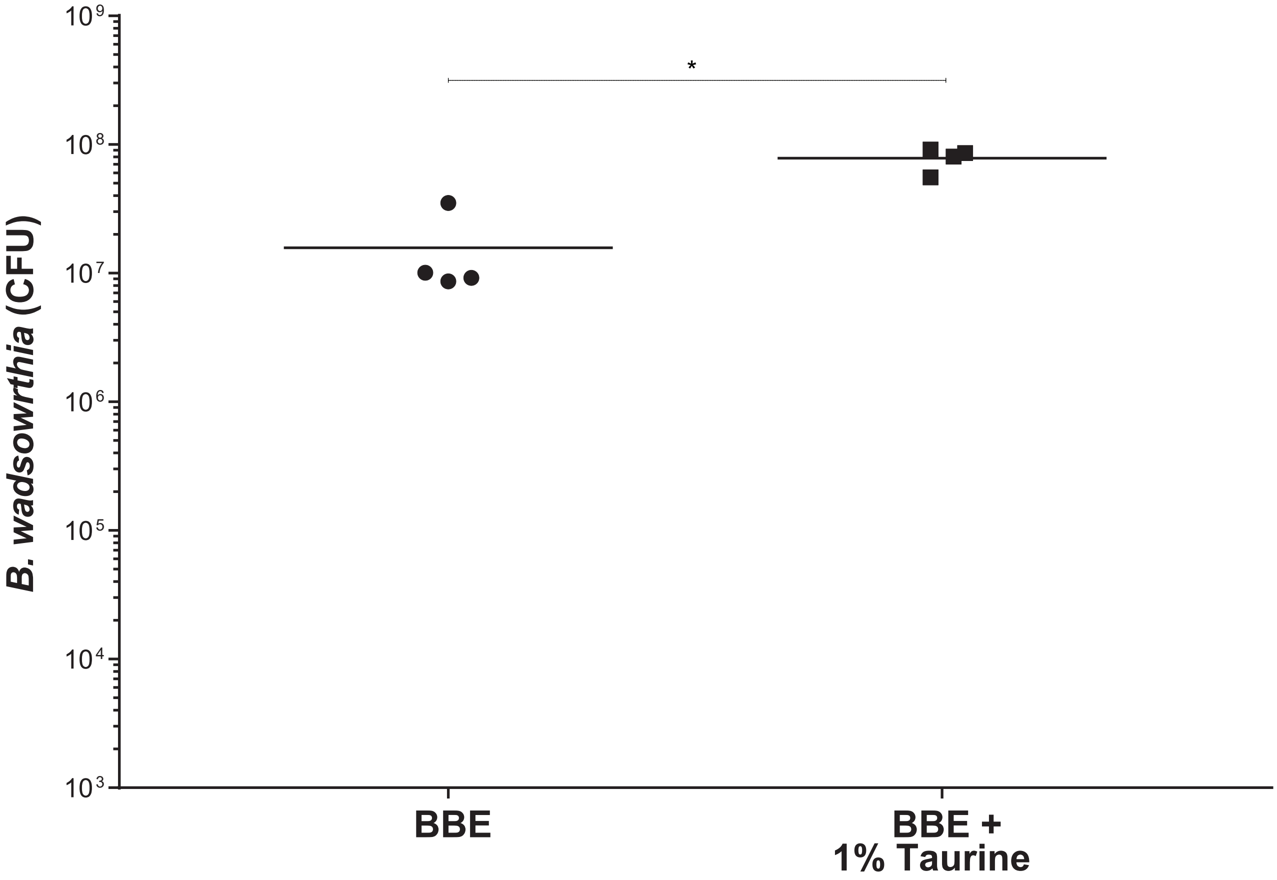

Supplementary Figure 8. *B. wadsworthia* growth in the presence of 1% Taurine. *B. wadsworthia* growth in BBE media supplemented or not with 1% taurine. Statistical comparison was performed by non-parametric Mann-Whitney test. (*$p<0.05$).

Supplementary Figure 9

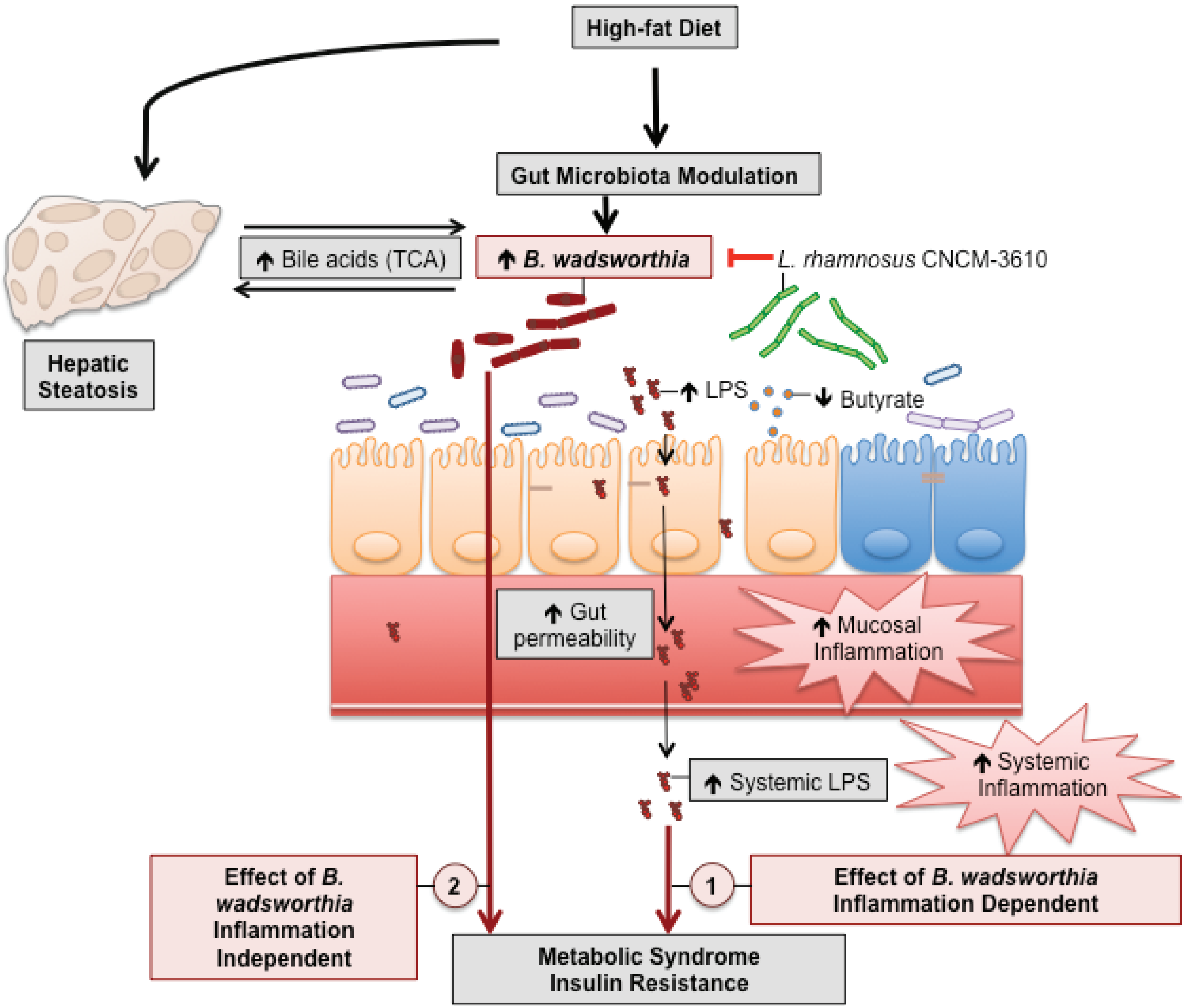

Supplementary Figure 9. Working hypothesis.
