# Supplementary material for: Bilophila wadsworthia aggravates high fat diet induced metabolic dysfunctions in mice
Source: Nat Commun. 2018 Jul 18;9:2802. doi: 10.1038/s41467-018-05249-7 (PMC6052103; doi:10.1038/s41467-018-05249-7)
Supplement: Supplementary file 3 — Description of Additional Supplementary Files [file 41467_2018_5249_MOESM3_ESM.pdf]

Description of Additional Supplementary Files

File Name: Supplementary Data 1
Description: Table of differentially expressed host genes between mice with CD and HFD.

File Name: Supplementary Data 2
Description: Host pathways with significantly altered activation between studied groups.

File Name: Supplementary Data 3
Description: Microbiota pathways with significantly altered activation between studied groups.
